# Initial investigation of free-breathing 3D whole-heart stress myocardial perfusion MRI

**DOI:** 10.21542/gcsp.2020.38

**Published:** 2020-12-31

**Authors:** Merlin J. Fair, Peter D. Gatehouse, Eliana Reyes, Ganesh Adluru, Jason Mendes, Tina Khan, Ranil de Silva, Rick Wage, Edward V.R. DiBella, David N. Firmin

**Affiliations:** 1 Cardiovascular Research Centre, Royal Brompton Hospital, London, UK; 2 National Heart & Lung Institute, Imperial College London, London, UK; 3 Department of Radiology and Imaging Sciences, University of Utah, Salt Lake City, UT, USA

## Abstract

**Objective:** Myocardial first-pass perfusion imaging with MRI is well-established clinically. However, it is potentially weakened by limited myocardial coverage compared to nuclear medicine. Clinical evaluations of whole-heart MRI perfusion by 3D methods, while promising, have to date had the limit of breathhold requirements at stress. This work aims to develop a new free-breathing 3D myocardial perfusion method, and to test its performance in a small patient population.

**Methods:** This work required tolerance to respiratory motion for stress investigations, and therefore employed a “stack-of-stars” hybrid Cartesian-radial MRI acquisition method. The MRI sequence was highly optimised for rapid acquisition and combined with a compressed sensing reconstruction. Stress and rest datasets were acquired in four healthy volunteers, and in six patients with coronary artery disease (CAD), which were compared against clinical reference information.

**Results:** This free-breathing method produced datasets that appeared consistent with clinical reference data in detecting moderate-to-strong induced perfusion abnormalities. However, the majority of the mild defects identified clinically were not detected by the method, potentially due to the presence of transient myocardial artefacts present in the images.

**Discussion:** The feasibility of detecting CAD using this 3D first-pass perfusion sequence during free-breathing is demonstrated. Good agreement on typical moderate-to-strong CAD cases is promising, however, questions still remain on the sensitivity of the technique to milder cases.

## Introduction

Impaired myocardial perfusion under stress is a consequence of several diseases, most prominently coronary artery disease where such impairment occurs early in the ischaemic cascade.^1^ Clinical testing for impaired myocardial perfusion by examining dynamically contrast-enhanced myocardial cardiovascular magnetic resonance (CMR) is well established,^2^ following its first demonstrations in 1990.^3^ Sometimes known as dynamic contrast enhancement (DCE), stress CMR myocardial first-pass perfusion (FPP) during the rapid bolus injection of gadolinium-based contrast agent (GBCA) demonstrates high diagnostic accuracy^4^ and compares favourably with nuclear medicine as a “gate-keeper” to invasive coronary x-ray angiography.^5,6^

In clinical CMR perfusion, myocardial T1-weighted images are acquired rapidly within each cardiac cycle tracing the arrival of contrast agent causing intensification of the myocardial image pixels, exposing perfusion abnormalities as regions of myocardium that brighten more slowly than normally perfused remote myocardium. This real-time bolus-tracking application demands “instant” imaging of multiple slices during each R-R interval, potentially with tachycardia during stress testing, which prevents MRI acquisition of fine image resolution throughout the entire myocardium during each cardiac cycle. Typically in routine clinical CMR FPP, three short-axis slices per cycle are acquired, as greater slice coverage requires some trade-offs. The limited myocardial coverage by FPP is seen as a weakness, and its extension to cover more myocardium^7^ may increase diagnostic confidence and potentially enable assessment of total myocardial ischemic burden. This extension of FPP is known as “3D FPP” because it is inherently a whole-volume acquisition, rather than multiple seperately acquired “2D” slices that have timing and cardiac phase differences.

3D FPP presents enormous technical challenges to implement. Here a brief review of previous 3D FPP technical approaches is made, prior to a summary of clinical evaluations of 3D FPP below. Most 3D FPP CMR work has used “undersampling” methods (Cartesian k-t undersampling)^8–10^ that make the fundamental assumption of limited respiratory motion and therefore limited, or at least gradual, changes during FPP in most of the field of view (FOV) outside the heart.^11^ These undersampling methods in 2D have been extended to improve tolerance to respiratory motion^12,13^ which nonetheless remains a fundamental limitation in this class of undersampling. Several other technical approaches for extending FPP to whole-heart coverage with 3D imaging have been proposed.^14^ By virtue of their repeated sampling of the strongest raw-data region (central k-space data sampling), radial acquisitions strongly support the undersampling required for 3D-FPP at an acceptable shot duration (where a shot is defined as the duration of data acquisition within a cardiac cycle) because they maintain reconstruction quality even after high levels of angular undersampling.^15,16^ This undersampling robustness, combined with the method known as “compressed sensing” (CS),^17^ potentially allows radial sequences to achieve high acceleration factors while retaining image quality (e.g.,^18^).

Most crucially for the aim of this paper, “radial” acquisitions of the MRI raw-data are also well-known for their tolerance to movement of the heart during the 3D shot (“intra-shot motion”),^19–21^ including cardiac motion and any intra-shot respiratory motion. In addition, 2D radial acquisitions have also been shown to potentially reduce the prevalence and extent of “dark-rim artefacts”^22^ which are sometimes difficult to discriminate from true perfusion abnormalities. One particular variant of radial 3D imaging, known as the stack-of-stars (SOS) sampling method, is a hybrid radial-Cartesian sampling strategy. By sampling in a Cartesian pattern along the left ventricular long axis, where the required resolution is typically coarser, this method therefore avoids the slower acquisition associated with fully radial 3D sampling. This SOS approach has been previously applied in 3D-FPP,^23–26^ and similarly for hybrid spiral-Cartesian 3D-FPP.^27^ Minimisation of the shot duration has needed particular focus because 3D imaging demands a substantial increase in shot duration compared to each slice by 2D-FPP. However, *in vivo* testing at stress is required to evaluate how these acquisitions cope with the increased heart-rates and respiratory motion. Furthermore, while imaging quality and reconstruction effects have been investigated to some extent, the diagnostic capability of 3D-SOS-FPP is unknown without testing in clinical populations against established diagnostic methods.

In clinical trials, whole-heart FPP by breath-held (Cartesian k-t) 3D imaging has shown high accuracy compared with quantitative coronary angiography (QCA)^28^ and compared well with fractional flow reserve (FFR) at two centres.^29^ The measurement of myocardial ischemic burden from 3D-FPP was also investigated, comparing well against that obtained by myocardial perfusion scintigraphy.^30^ Multi-centre evaluation of the same CMR method against QCA and FFR^10^ also showed strong performance. Radial methods such as SOS and reconstruction with compressed sensing methods, which should benefit from an extension to 3D, have so far undergone significantly less investigation in clinical populations for 3D-FPP.

Therefore, to investigate the capability of a free-breathing 3D-SOS-FPP sequence, clinical testing is required at stress and in patient populations with known CAD. In this work, a 3D-SOS-FPP sequence was optimised to acquire datasets at stress and rest in healthy volunteers and patients with suspected CAD. The patients also underwent at least one standard clinical investigation (SPECT MPS, X-ray angiography, CMR 2D-FPP) as a reference for the comparison of any detected induced perfusion defects. This work aims to investigate the capability of free-breathing compressed-sensing 3D-SOS-FPP to identify stress-induced myocardial perfusion defects.

## Methods

### Subject recruitment

Informed consent under ethical approval was acquired from all ten subjects. All of the healthy volunteers and CAD patients underwent the same stress/rest scanning procedure.

Four volunteers with no suspected CAD or other cardiac conditions were recruited. These acquisitions aimed to provide initial validation of the sequence acquisition under stress, and were performed using an identical procedure as planned for the patients with clinical evidence of CAD.

Six patients were recruited for the stress/rest 3D-SOS-FPP protocol (in a seventh patient, scanning was abandoned due to an adenosine-induced ECG rhythm disturbance before the stress scan could be acquired). The patients were selected after clinical referral for MPS, X-ray catheter angiography, or conventional CMR 2D-FPP, with a range of coronary artery disease severity, with the aim of testing the response range of 3D-SOS-FPP compared to clinical reference methods. Exclusion criteria before invitation to participate were any contraindications to MRI, adenosine or GBCA, and also arrhythmia. The patient history and information was reviewed by a clinician to check suitability to participate.

### Subject scanning protocol

All subjects were positioned supine and scanned using a standard clinical spine coil and anterior 18-channel coil on a Siemens Skyra 3T “XQ-engine” (Siemens Medical Systems, Erlangen, Germany) with the standard clinical ECG-gating module attached.

The following stress protocol was implemented to ensure consistent scanning of each subject with the 3D-SOS-FPP sequence. After initial “scout” and LV cine acquisitions to locate the heart, the stress 3D-SOS-FPP acquisitions were planned with the LV centred in the volume to be imaged, and a trigger delay was adjusted to time the central raw data (“k-space”) of the 3D “shot” acquisition optimally against LV myocardial motion. This was done by accurately timing the acquisition for during the end-systolic pause, which was chosen due to the shortening of diastasis at higher heart rates,^31^ i.e., scanning under pharmacologically-induced stress. The end-systolic pause also enabled a smaller long-axis FOV for the 3D-SOS-FPP.^14^ Magnetic field shimming (second-order) and reference frequency adjustment were applied, by the conventional manufacturer’s methods.

In each patient, a trial run of the planned 3D-SOS-FPP sequence was acquired and reconstructed, to ensure correct planning (e.g., position and coil selections), and that no gradient system errors or nerve stimulation warnings occurred, as these can depend on cardiac orientation. The trial also confirmed that the ECG was supporting reliable R-wave triggering during sequence operation, essential to withstand additional stress-related challenges to this complex device. To enable immediate examination of the trial images, these were reconstructed by a simplified method (a non-iterative gridding operation) to produce low-quality but near-instantaneous images.

Following an acceptable 3D-SOS-FPP trial result, administration of adenosine (140 μ g/kg/min) began, with a clinician monitoring the subject’s cardiac rhythm, heart-rate, blood pressure and symptoms (such as breathlessness and angina) (see Discussion regarding breathing motion). Approximately six minutes of infusion was used to ensure onset of the stress condition, although the subject’s physiological responses were also considered in determining when a stable degree of stress had been reached. No specific breathing instructions were given, except to try and avoid coughing or bulk motion during the scanning session.

During the onset of stress, a single mid-LV SAX cine was acquired during free-breathing by taking highly accelerated multiple averages over 12 cardiac cycles. Final adjustments could then be made to the 3D-SOS-FPP trigger delay, based on any changes in the time to end-systole observed from the cine images. As this adjustment procedure could require up to two minutes, it was necessary to proceed with this stress cine acquisition about half-way through the initial infusion.

The 3D-SOS-FPP sequence was applied during free-breathing during the continued adenosine infusion, capturing the first-pass of 0.1 mmol/kg antecubitally power-injected gadobutrol (Gadovist 1M, Bayer) at 3 ml/s, with 20 ml saline flush at 5 ml/s. After completion of the 3D-SOS-FPP scan, adenosine infusion was halted and the subject was checked for the dissipation of its effects. After >20 minutes post-injection of the first dose of gadolinium, the 3D-SOS-FPP sequence was repeated as before, without adenosine infusion, to provide the “rest” dataset.

Technical details of the whole-heart stack-of-stars first-pass-perfusion (3D-SOS-FPP) method (S1 - Appendix A1), its optimisation to allow a shot duration of 188 ms (S1 - Appendices A2 and A3) and its image reconstruction (S1 - Appendix A4) are included in the Appendices and^32^.

### Clinical reference methods

The healthy volunteers had no clinical reference tests, on the assumption of normal stress perfusion.

In four of the six patients, technetium-99m tetrofosmin (Myoview, GE Healthcare, USA) SPECT MPS was performed with a standard clinical protocol. Three of these four patients were scanned on a D-SPECT (Spectrum Dynamics, Israel) solid-state gamma camera and the fourth on a CardioMD (Philips, Netherlands) conventional gamma camera. All were imaged with a 1-day stress and rest protocol, using a 250 MBq dose at stress and a 750 MBq dose at rest. Adenosine or regadenoson was used as the primary vasodilator (depending on clinical indication) and the technetium-99 m tetrofosmin was injected three minutes into the infusion of adenosine, or 90 seconds after the regadenoson injection. Image acquisition was started 60 minutes after the injection of the tracer and at least three hours were left between the tracer injections for the stress and rest acquisitions. On the D-SPECT camera dual supine and upright stationary acquisitions were acquired (to aid artefact detection), for approximately six minutes at stress and three minutes at rest. The CardioMD scanner, equipped with an all-purpose collimator, acquired 64 projections over 180 degrees while the patient was supine, in approximately 20 minutes. A 20% symmetrical energy window was used at 140 keV. Attenuation correction was applied.

In the other two of the six patients, clinical reports from standard stress and rest 3-slice 2D-FPP CMR were the reference test, although obtained at 3.5 and 4.5 years before the 3D-SOS-FPP (see Discussion). At the same 3T scanner, three eight mm short-axis slices were acquired per cardiac cycle in basal to apical order at four mm and eight mm gaps during the antecubital injection of 1 M GBCA (gadobutrol) at 3 ml/s followed by 20 ml saline flush, by standard clinical parameters (S1 - Appendix A5).

Coronary X-ray angiography (CXA) was additionally available in one patient, obtained 2 years before the 3D-SOS-FPP (see Discussion). The CXA was undertaken via the femoral approach using standard coronary catheters, feeding them to the origins of the left and right coronary systems sequentially. Following this, a small amount of radiopaque contrast was injected in the coronary during X-ray projection imaging, repeating at different projection angles to allow the visualisation of the coronary from multiple 2 dimensional planes. Once all the images had been obtained of the native circulation, any additional grafts were visualised using specific catheters.

### Data analysis

Only a small number of healthy volunteers and CAD patients volunteered for stress imaging with contrast agent for this research. With this small study, it was inappropriate to apply statistical analyses of any quantitative measures of induced perfusion defects (e.g., between 3D-SOS-FPP and MPS). Therefore, examination was made on a case-by-case basis of each 3D-SOS-FPP dataset to identify potential perfusion defects. In the CAD patients, the 3D-SOS-FPP datasets were compared with the diagnoses already reported clinically from the reference tests (MPS or 2D-FPP) determining which myocardial segments were considered likely to demonstrate stress-induced perfusion defects, and whether those were expected to be mild, moderate or severe. The 3D-SOS-FPP was examined by two cardiologists with approximately 10 and 30 years’ experience including MPS, CXA and (since approx 2000) by CMR. The readers were blinded to any other diagnostic information. A consensus was agreed in the case of different opinions.

## Results

Results from all ten subjects (four healthy volunteers and six volunteer patients) are presented. Except for a seventh patient as explained in Methods, there were no other attempts to perform stress 3D-SOS-FPP that were excluded, for example due to poor image quality; the 3D-SOS-FPP was simple and reliable in application with little sensitivity to magnetic field shimming or reference frequency adjustment.

### Healthy volunteers

**Figure 1.**
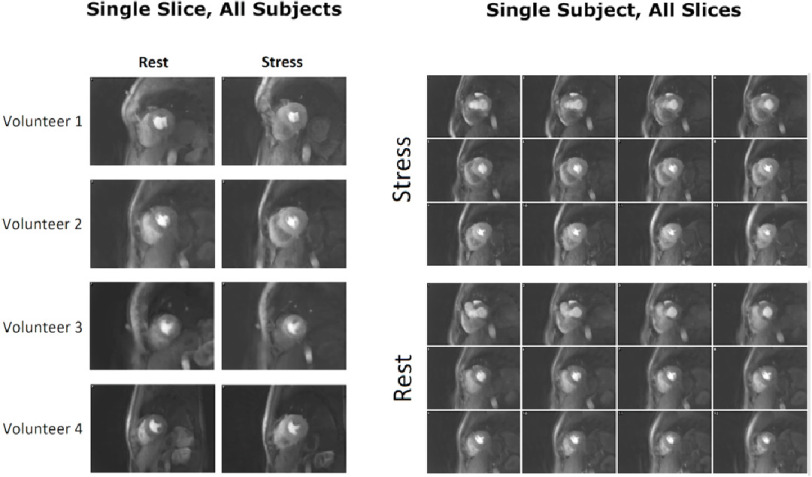
Healthy volunteer overview. An overview of the four healthy volunteer subjects, demonstrating a single central slice from 3D-SOS-FPP at a single time point of GBCA arrival in the left ventricle for each subject (left), and for 12 reconstructed five mm slices from base to apex of a single temporal frame (right). The remaining 4 slices are not shown as those are at the edges of the slab selection and partition-encoding direction as is usual in 3D MRI. All of the images from a 3D-SOS-FPP shot are at the same cardiac timing (see Discussion) unlike the multiple slices of 2D-FPP.

**Figure 2.**
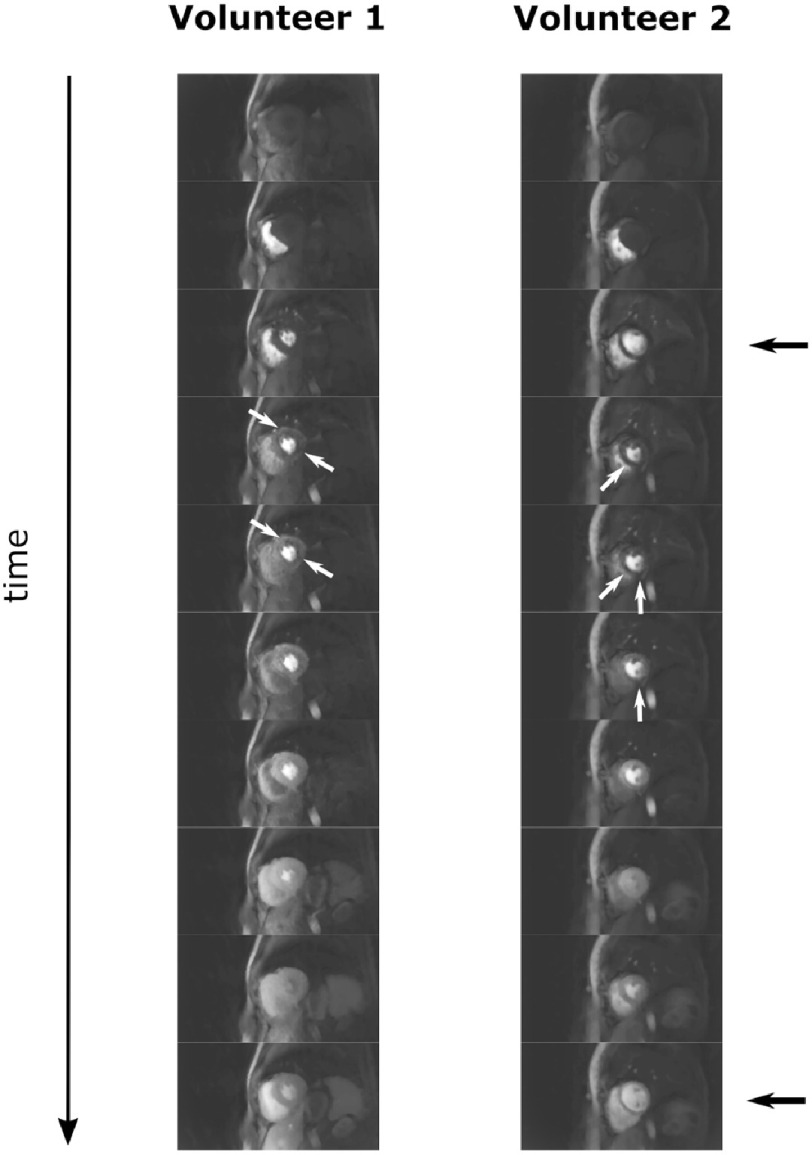
GBCA arrival dynamics in healthy volunteers. 3D-SOS-FPP frames are selected to demonstrate the arrival of the GBCA (top to bottom) in two healthy volunteers, at stress. Both datasets demonstrate myocardial artefacts with a dark-patch appearance (short white arrows), which occur during the arrival of the GBCA into the LV myocardium but quickly dissipate, apparently as the myocardium brightens. The dataset on the right also contains frames where mis-triggering has occurred (short black arrows).

All four healthy subjects were successfully scanned (Figure 1), and the reconstructions tolerated the wide range of respiratory motions at both rest and stress. At stress imaging, the heart rates reached up to 97 bpm. Good coverage of the LV myocardium was achieved in all cases. Promisingly, no persistent dark regions were seen in their myocardium at stress or at rest. Transient myocardial dark artefacts were seen in 2 of the healthy volunteer datasets during the GBCA arrival (Figure 2). The artefacts remain for 2–3 frames before dissipating as the myocardial intensity increases and the LV bloodpool becomes less bright, as with the well-known dark-rim artefact (DRA) in 2D-FPP.^33^ One of these datasets suffered from poor ECG-triggering at stress (Figure 2, right column).

### CAD patients

Six CAD patients completed the stress protocol and are labelled Patient 1–6. Movies showing the first-pass perfusion in all slices obtained by 3D-SOS-FPP for all 6 patients are available online (S2 - Movies M1-M12). Overall, the respiratory motion appears relatively shallow, despite the absence of any breathing instructions given to subjects. The results are summarised in Tables 1 and 2 comparing clinical reference data against the 3D-SOS-FPP findings.

**Table 1:** Comparison of clinical reference data with whole-heart 3DFPP.

**PatientNumber**	**Clinical reference on stress-induced defects**	**3D-SOS-FPP finding**
1	Basal septal/infero-septal severe. Apical infero-lateral mild.	Basal septal/infero-septal severe. Apical infero-lateral mild.
2	Basal and mid, inferior/ infero-lateral moderate.	Basal and mid, inferior/ infero-lateral moderate.
3	Basal and mid, antero-septal mild.	Normal.
4	Apical, anterior, mild.	Normal.
5	Mid and apical, antero-septal, mild. Basal, inferior/infero-septal/infero-lateral, mild.	Mid and apical, antero-septal, mild.
6	Basal and mid, inferior/infero-septal, mild.	Normal.

**Notes.**

The terms mild/moderate/severe are defined by the impression of the defect’s combined transmurality, depth of intensity and temporal persistence.

**Table 2:** Total number of identified myocardial segments.

	**Severe**	**Moderate**	**Mild**
Clinical Reference	2	3	12
3D-SOS-FPP	2	3	3

**Notes.**

Total number of myocardial segments with identified perfusion abnormalities across all patients, for the reference method and for 3D-SOS-FPP, separated by the severity of the perfusion abnormality.

#### Severe ischaemia detection

**Figure 3.**
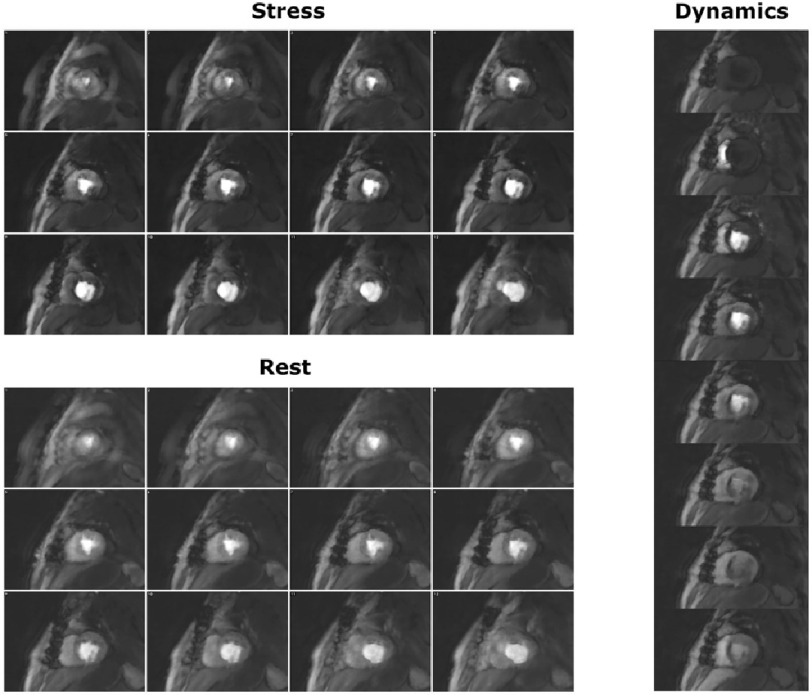
FPP images for Patient 1. The stress (top) and rest (bottom) 3D-SOS-FPP slices, for one perfusion frame. Hypointense myocardial regions can be seen, including in locations that match with the MPS data. Examination of the temporal dynamics (right, and S2 movie M1) is required to identify the persistence of these hypointense regions.

**Figure 4.**
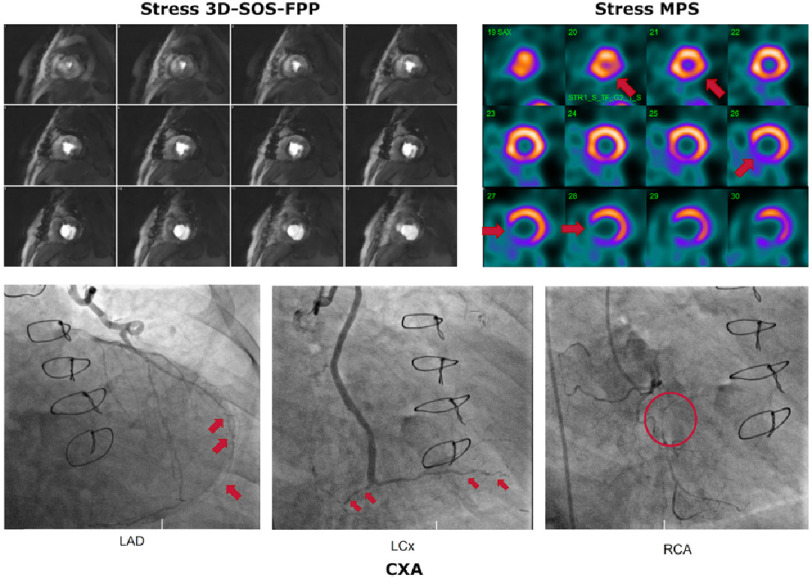
Comparison of 3D-SOS-FPP images with MPS and CXA for Patient 1. The patient was judged to have severe inducible ischaemia. The stress 3D-SOS-FPP images (left) are ordered so as to be as close as possible to their stress MPS equivalent (right). A strong septal/infero-septal defect can be seen in the base-to-mid slices, as well as a milder infero-septal defect in the apical slices. CXA of the left anterior descending (LAD), left circumflex (LCx) and right coronary (RCA) arteries are shown below. For the LAD, examples of the diffuse disease of the vessels are marked by red arrows. Sternal wire loops are visible, which did not disrupt the 3D-SOS-FPP method as it was based on short TE and spoiled-gradient-echo imaging. In the LCx, examples of distal disease of the vessel are marked by red arrows. For the RCA, an area of occlusion is marked by the red circle.

Patient 1 was classified by MPS as having severe stress-induced ischaemia. The MPS images at stress showed a strong septal/infero-septal defect basally and a milder infero-lateral defect apically, that did not occur at rest. The stress 3D-SOS-FPP images (Figure 3) showed clear, persistent hypointense regions, closely matching with MPS (Figure 4), which were not present at rest.

The strength and persistence of the hypointense regions can clearly be seen during the GBCA first-pass (Figure 3 and S2 movies M1 and M2). Some additional transient hypointense regions during the GBCA arrival into the LV myocardium were similar to the artefacts identified in the healthy volunteers.

This patient had also undergone recent CXA, which confirmed that the patient had substrates for ischaemia in all three coronary territories (Figure 4) agreeing with 3D-SOS-FPP.

#### Moderate ischaemia detection

**Figure 5.**
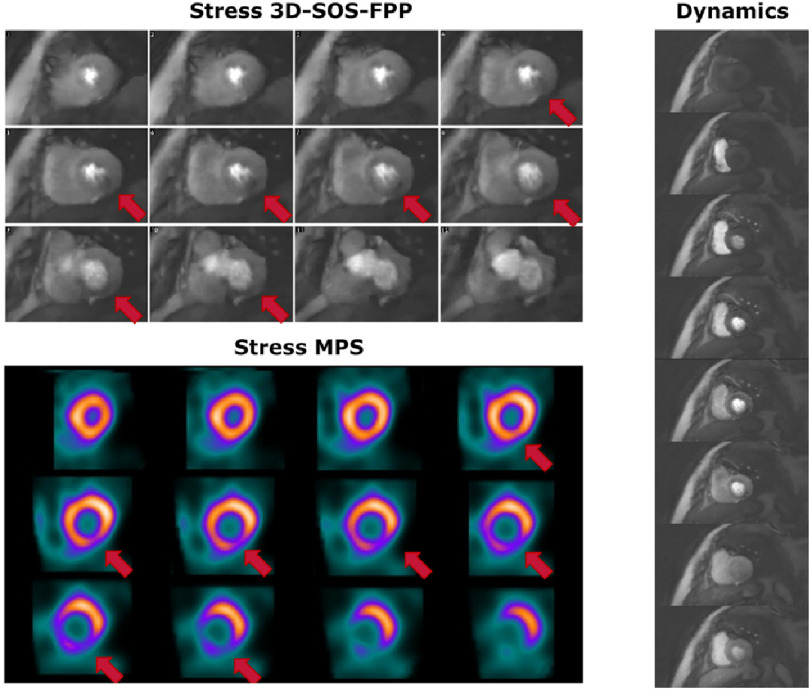
Comparison of 3D-SOS-FPP images with MPS for Patient 2. The patient was judged to have moderate inducible ischaemia. Stress 3D-FPP (top) and stress MPS (bottom) are shown. Red arrows mark potential defects in both datasets. For a single slice of the 3D-SOS-FPP dataset, multiple frames for the first-pass of the GBCA are displayed (right). Transient dark myocardial artefacts occur in the anterior and antero-septal regions of the myocardium but do not show the temporal persistence of the dark inferior myocardial region which correlates with the MPS finding.

From the MPS, patient 2 was classed as moderate and showed an apparent inferior/infero-lateral stress-induced defect by 3D-SOS-FPP in the basal to mid-ventricular images, which matched well with the MPS scan (Figure 5). Again, some transient dark myocardial artefacts occurred during GBCA arrival (S2 movies M3 and M4). However, this hypointensity persisted only in regions of the myocardium matching spatially with the MPS defects.

#### Mild ischaemia detection

**Figure 6.**
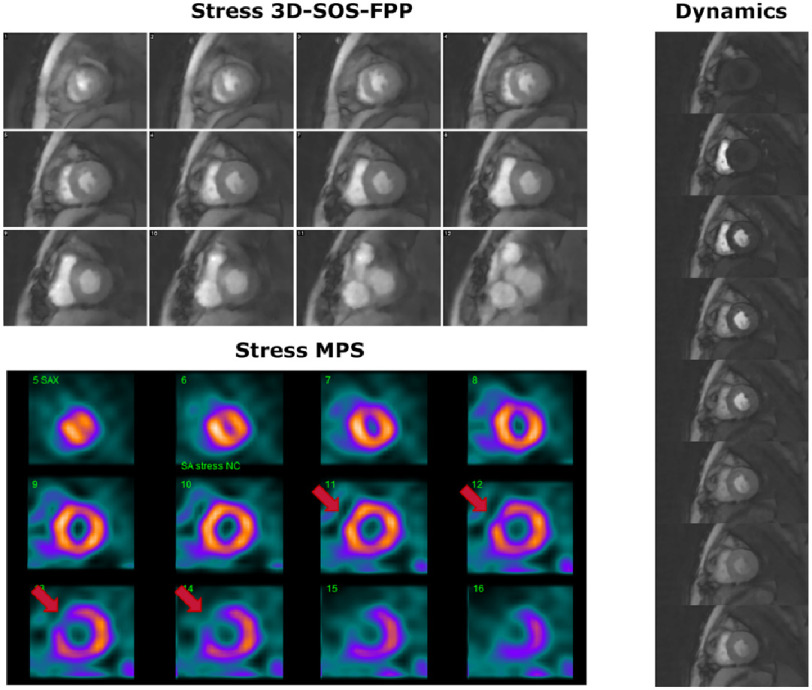
Comparison of 3D-SOS-FPP images with MPS for Patient 3. The patient was judged to have mild inducible ischaemia. Stress 3D-SOS-FPP (top) and stress MPS (bottom) are shown for Patient 3. Red arrows mark potential defects in the MPS dataset. For a single slice of the 3D-SOS-FPP dataset, multiple frames for the first-pass of the GBCA are displayed (right). No persistent stress defect could be seen.

In patient 3, with mild CAD, no agreement was found between the stress FPP and MPS images (Figure 6 and S2 movies M5 and M6). The stress FPP dataset showed a smooth, consistent uptake of GBCA quite uniformly over the LV, with no obvious hypointense regions to match the antero-septal defect suggested by the MPS.

In patient 4, (S2 movies M7 and M8) the 3D-SOS-FPP images were unclear compared to the other datasets, caused by mistriggering of the ECG during stress imaging. The very mild defect detected by MPS was not visible on the stress 3D-SOS-FPP, with increased artefacts in the myocardium during contrast arrival.

In patient 5, agreement was found for the mid and apical slices showing an antero-septal mild perfusion defect in 3D-SOS-FPP (S2 movies M9 and M10) as seen on 2D-FPP (S2 movie M13); these were present in the stress scan but not in the rest scan. However, a mild basal inferior perfusion defect noted on 2D-FPP was not found in the 3D-SOS-FPP.

In patient 6, no agreement was found between 3D-SOS-FPP (S2 movies M11 and M12) and 2D-FPP (S2 movie M14). A basal inferior mild stress perfusion defect was found in 2D-FPP stress first-pass imaging and not in the resting 2D-FPP first-pass. A mild subendocardial defect was seen at stress in 3D-SOS-FPP, and was also seen during rest 3D-SOS-FPP imaging; this defect did not correspond to any late-enhancement of the LV myocardium.

## Discussion

### Diagnostic capability

The healthy volunteers showed no persistent hypointense myocardial regions during FPP, as consistent with normal myocardial perfusion at stress and at rest.

**Figure 7.**
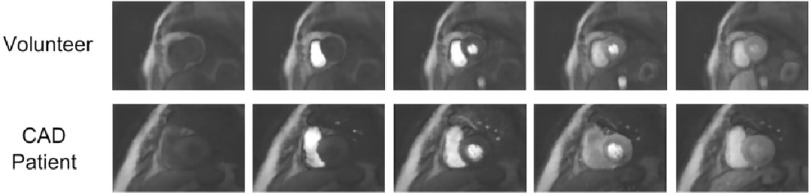
Volunteer and CAD patient dynamics comparison. Multiple frames of a single slice taken from a stress dataset in a healthy volunteer (top) and CAD patient (bottom), showing the dynamics of the 3D-SOS-FPP and persistence of a genuine stress-induced defect in the inferior wall of the LV.

In this small sample size of six CAD patients, as summarised by Tables 1 and 2, the overall performance in comparison to clinical reference data was mixed. The only severe CAD case, patient 1, was the easiest in which to determine the presence of a perfusion defect, with strong agreement of 3D-SOS-FPP with MPS and CXA. The longer persistence and greater transmurality of the stress-induced defects in this case enabled clear differentiation from transient myocardial dark artefacts. Figure 7 shows a series of images during first-pass demonstrating the persistence of myocardial perfusion defects in this patient compared to the transient artefacts in a normal subject.

The moderate to mild cases presented a greater challenge to ischaemia detection, and the results Tables 1 and 2 show clearly that 3D-SOS-FPP correlated less reliably with MPS and 2D-FPP in the mild cases. While the images of Patient 4 were degraded by the mis-triggering which probably impeded detection of the small expected defect, Patients 3, 5 and 6 showed high image quality by 3D-SOS-FPP but the stress images appeared to miss some perfusion defects identified by MPS or 2D-FPP, raising potential questions over the accuracy of the technique. It is possible the undersampling degraded the true resolution of the acquired data severely enough to conceal a small defect, which the STCR reconstruction was unable to restore on assembling the full angular resolution (details in S1 - Appendix A4). Alternatively, it is conceivable that the reference method was inaccurate or overestimated the extent of a perfusion deficit. Some differences between 3D-SOS-FPP and the reference clinical data might arise from the long interval since the clinical test (up to 5 years). Unfortunately, CXA was not available in these patients to examine the extent of CAD.

Nevertheless, these acquisitions provide initial tests to detect inducible myocardial perfusion defects over the entire LV with free-breathing 3D-SOS-FPP and its compressed-sensing reconstruction methods, in agreement with MPS and CXA in cases with severe and moderate CAD. This work may provide insight into the clinical feasibility of free-breathing whole-heart 3D-SOS-FPP in the context of other approaches to respiratory motion in advanced subsampling FPP sequences.^12,13^

### Image quality

For the previously published clinical stress validations of 3D-FPP (Cartesian k-t techniques), breath-holding was required for accurate reconstruction of the strongly reduced number of acquired k-space lines. Synchronising this breath-hold request with GBCA first-pass arrival into the myocardium could reduce reliability as co-operation under stress is often more difficult. The progression to free-breathing evaluated in this work is therefore considered to an important aim for realistic clinical application. At the onset of adenosine stress, a feeling of breathlessness is quite common and naturally disturbs regular breathing. However, the perfusion scanning was obtained after approximately six minutes of infusion when a steady degree of stress was considered established, and breathing had stabilised. There was no request for breath-holding or shallow breathing in this work, and therefore no increased risk that patients might “gasp” for a deep breath abruptly during the perfusion acquisition as a consequence of breathlessness arising after following such instructions. While SOS is more compatible than Cartesian methods with “undersampling” of the MRI raw data, free-breathing acquisitions nevertheless increase the burden on the reconstruction technique through increased inconsistency in the undersampled data (essentially this increases the challenge placed on the mathematical estimation of the unrecorded data).

Despite this, degradation of the image quality by respiratory motion was rarely obvious, and image quality did not break down completely in any of this work, but the impact on the clarity and temporal fidelity of the datasets remains less clear. The optimisation of weighting parameters balancing between fidelity and potential smoothing required careful attention during initial development. After that initial “set-up” work, individual adjustments of reconstruction parameters per case were not performed (S1 - Appendix A4). Ideally, further work is required to explore the reconstruction accuracy and resolution loss of a variety of STCR reconstruction approaches, during FPP perfusion, with and without respiratory motion.

A notable advantage of 3D-FPP not widely recognised is that all the images of the heart are at the same cardiac phase, simplifying reading of the results and ensuring that through-slice motion between the timings of separate slices does not confound exactly what tissue was being imaged; furthermore, any cardiac-motion related dark rim artefacts (DRA)^34^ would at least appear the same across all the myocardial coverage of the 3D images, unlike the 2D multislice FPP approach where the change in cardiac phase between slices can cause confusion by generating strongly differing “intra-shot” cardiac-motion induced dark rim artefacts in each myocardial slice.

Dark transient myocardial artefacts are common in clinical 2D-FPP and with experience can be identified, aided by their transience and correlation with blood-to-myocardium image contrast. However, their frequent occurrence remains troublesome, as they are difficult to discriminate from mild rapid-filling sub-endocardial perfusion defects. Initial simulations and phantom investigations for 3D-SOS-FPP explored potential causes of transient dark artefacts in 3D-FPP,^35,36^ with the aim of reducing their presence or severity.

### Other considerations

In keeping with the aim for a simple reliable method for clinical adoption, 3D-SOS-FPP required only selection of its slab position and the trigger delay for each patient. The only increase in complexity over a typical clinical 2D multi-slice FPP protocol was the inclusion of the additional cine acquired at stress to help optimise the cardiac end-systolic timing of the 3D acquisition. Based upon this stress cine acquisition, adjustments to the 3D-SOS-FPP trigger delay were made in the 7 of the 11 patients. The changes in timing were small (≤20 ms) and it is likely that this adjustment was not essential.

The radial resolution (pixel size) in 3D-SOS-FPP was nominally finer than for typical clinical CMR FPP acquisitions, although the true in-plane resolution delivered by the reconstruction (STCR, S1 - Appendix A4) in free-breathing is difficult to characterise. The through-plane resolution of 3D-SOS-FPP is slightly coarser than the 8.0 mm commonly used in clinical CMR 2D-FPP where apical slices show through-plane partial-volume effects. Increased myocardial coverage was achieved comparative to typical multi-slice clinical CMR 2D FPP in every cardiac cycle.

Cardiac motion during the shot did not appear to degrade endocardial border clarity to the degree that one might expect. The 188 ms shot duration is comparable to the previous clinical validations of Cartesian 3D-FPP sequences, where high diagnostic accuracy has been achieved with similar shot durations at systole (e.g.,^10^). Other image artefacts were infrequent: off-resonance artefacts were not discernible, matching initial phantom investigations; this reliability is well understood in MRI and arose from the short duration of each sampling of rawdata (“short k-space path”) within each shot and was a strong advantage for trials involving stress and contrast agent injections.

## Conclusions

The technical reliability necessary for routine clinical use of 3D-SOS-FPP at stress and rest during free-breathing has been demonstrated. This included an initial demonstration of inducible perfusion defects by 3D-SOS-FPP employing compressed-sensing reconstruction and acquired in free-breathing. The diagnostic capability of this technique is still uncertain following this first study, which had a small sample size. Within the six patients examined, all perfusion deficits judged as severe or moderate from a clinically accepted reference method were also identified through the 3D-SOS-FPP method. However, the stress results in 3D-SOS-FPP did not match in the majority of the mild deficits that were identified through the other reference methods. Furthermore, the presence of dark transient artefacts was an issue which requires further work. The potential feasibility of using free-breathing non-Cartesian 3D-SOS-FPP methods for detecting severe perfusion abnormalities has been shown, while this work also exposes a previously neglected potential issue in detecting mild perfusion abnormalities with such methods.

**SUPPORTING INFORMATION**

S1 –  Appendices

Available at https://globalcardiologyscienceandpractice.com/index.php/gcsp/article/view/455/396

A1–Whole heart stack-of-stars first-pass-perfusion (3D-SOS-FPP) method

A2–Shot duration minimisation

A3–RF pulse optimisation

A4–STCR reconstruction and L-curve optimisation

A5–2D CMR FPP acqusition details

S2–Movie Files

Available at: https://www.dropbox.com/sh/is4srut8usx8rfh/AACZ1NSKoPbXxhAlf7ctE1pXa?dl=0

M1: 12 slices of a 3D-SOS-FPP acquisition at stress, in patient 1. A strong septal/infero-septal defect can be seen in the base-to-mid slices, as well as a milder infero-lateral defect in the apical slices.

M2: 12 slices of a 3D-SOS-FPP acquisition at rest, in patient 1. No persistent hypointense regions are visible.

M3: 12 slices of a 3D-SOS-FPP acquisition at stress, in patient 2. An inferior/infero-lateral stress-induced defect can be seen.

M4: 12 slices of a 3D-SOS-FPP acquisition at rest, in patient 2. No persistent hypointense regions are visible.

M5: 12 slices of a 3D-SOS-FPP acquisition at stress, in patient 3. No obvious hypointense regions are present in the uptake to match the antero-septal defect suggested by the MPS.

M6: 12 slices of a 3D-SOS-FPP acquisition at rest, in patient 3 No persistent hypointense regions are visible.

M7: 12 slices of a 3D-SOS-FPP acquisition at stress, in patient 4. Increased artefacts in the myocardium during contrast arrival, caused by mistriggering of the ECG during stress imaging. The very mild defect detected by MPS is not evident.

M8: 12 slices of a 3D-SOS-FPP acquisition at rest, in patient 4. No persistent hypointense regions are visible.

M9: 12 slices of a 3D-SOS-FPP acquisition at stress, in patient 5. Mid and apical slices show an antero-septal mild perfusion defect. However, a mild basal inferior perfusion defect noted on 2D-FPP is not apparent.

M10: 12 slices of a 3D-SOS-FPP acquisition at rest, in patient 5. No persistent hypointense regions are visible.

M11: 12 slices of a 3D-SOS-FPP acquisition at stress, in patient 6. A basal inferior mild stress perfusion defect found in 2D-FPP stress is not evident. An apparent mild subendocardial defect seen here at stress matches one seen in the rest 3D-SOS-FPP imaging; this defect did not correspond to any late-enhancement of the LV myocardium.

M12: 12 slices of a 3D-SOS-FPP acquisition at rest, in patient 6. An apparent mild subendocardial defect seen here did not correspond to any late-enhancement of the LV myocardium.

M13: 3 slices of a clinical 2D-FPP acquisition at stress (top) and at rest (bottom), in patient 5. A mild defect can be seen in each of the basal, mid and apical slices, at stress.

M14: 3 slices of a clinical 2D-FPP acquisition at stress (top) and at rest (bottom), in patient 6. A basal inferior mild stress perfusion defect is seen at stress and not in the resting first-pass.

## Acknowledgements

We would like to thank the staff of the Cardiac MRI unit of the Royal Brompton Hospital for their assistance with this work.

## References

[1] Nesto RW, Kowalchuk GJ (1987). The ischemic cascade: Temporal sequence of hemodynamic, electrocardiographic and symptomatic expressions of ischemia. Am J Cardiol.

[2] Skinner JS, Smeeth L, Kendall JM, Adams PC, Timmis A (2010). NICE guidance Chest pain of recent onset: assessment and diagnosis of recent onset chest pain or discomfort of suspected cardiac origin. Heart.

[3] Atkinson DJ, Burstein D, Edelman RR (1990). First-pass cardiac perfusion: evaluation with ultrafast MR imaging. Radiology.

[4] Kim WY, Danias PG, Stuber M, Flamm SD, Plein S, Nagel E (2001). Coronary magnetic resonance angiography for the detection of coronary stenoses. N Engl J Med.

[5] Schwitter J, Wacker C, Wilke N, Al-Saadi N, Sauer E, Huettle K (2012). Superior diagnostic performance of perfusion-cardiovascular magnetic resonance versus SPECT to detect coronary artery disease: The secondary endpoints of the multicenter multivendor MR-IMPACT II (Magnetic Resonance Imaging for Myocardial Perfusion Assessment in Coronary Artery Disease Trial). J Cardiovasc Magn Reson.

[6] Greenwood JP, Maredia N, Younger JF, Brown JM, Nixon J, Everett CC (2012). Cardiovascular magnetic resonance and single-photon emission computed tomography for diagnosis of coronary heart disease (CE-MARC): a prospective trial. The Lancet.

[7] Shin T, Hu HH, Pohost GM, Nayak KS (2008). Three dimensional first-pass myocardial perfusion imaging at 3T: feasibility study. J Cardiovasc Magn Reson.

[8] Vitanis V, Manka R, Giese D, Pedersen H, Plein S, Boesiger P (2011). High resolution three-dimensional cardiac perfusion imaging using compartment-based k-t principal component analysis. Magn Reson Med.

[9] Jogiya R, Kozerke S, Morton G, Silva KDe, Redwood S, Perera D (2012). Validation of Dynamic 3-Dimensional Whole Heart Magnetic Resonance Myocardial Perfusion Imaging Against Fractional Flow Reserve for the Detection of Significant Coronary Artery Disease. J Am Coll Cardiol.

[10] Manka R, Wissmann L, Gebker R, Jogiya R, Motwani M, Frick M (2015). Multicenter evaluation of dynamic three-dimensional magnetic resonance myocardial perfusion imaging for the detection of coronary artery disease defined by fractional flow reserve. Circ Cardiovasc Imaging.

[11] Tsao J, Boesiger P, Pruessmann KP (2003). k-t BLAST and k-t SENSE: Dynamic MRI with high frame rate exploiting spatiotemporal correlations. Magn Reson Med.

[12] Schmidt JFM, Wissmann L, Manka R, Kozerke S (2014). Iterative k-t principal component analysis with nonrigid motion correction for dynamic three-dimensional cardiac perfusion imaging. Magn Reson Med..

[13] Zhou R, Huang W, Yang Y, Chen X, Weller DS, Kramer CM (2018). Simple motion correction strategy reduces respiratory-induced motion artifacts for k-t accelerated and compressed-sensing cardiovascular magnetic resonance perfusion imaging. J Cardiovasc Magn Reson.

[14] Fair MJ, Gatehouse PD, DiBella EV, Firmin DN (2015). A review of 3D first-pass whole-heart myocardial perfusion cardiovascular magnetic resonance. J Cardiovasc Magn Reson.

[15] Adluru G, McGann C, Speier P, Kholmovski EG, Shaaban A, DiBella EVR (2009). Acquisition and reconstruction of undersampled radial data for myocardial perfusion magnetic resonance imaging. J Magn Reson Imaging.

[16] Uecker M, Zhang S, Frahm J (2010). Nonlinear inverse reconstruction for real-time MRI of the human heart using undersampled radial FLASH. Magn Reson Med.

[17] Lustig M, Donoho D, Pauly JM (2007). Sparse MRI: The application of compressed sensing for rapid MR imaging. Magn Reson Med.

[18] Usman M, Atkinson D, Odille F, Kolbitsch C, Vaillant G, Schaeffter T (2013). Motion corrected compressed sensing for free-breathing dynamic cardiac MRI. Magn Reson Med.

[19] Rasche V, Holz D, Köhler J, Proksa R, Röschmann P (1997). Catheter tracking using continuous radial MRI. Magn Reson Med.

[20] Uecker M, Zhang S, Voit D, Merboldt K, Frahm J (2012). Real-time Mri: recent advances using radial Flash. Imaging Med.

[21] Pflugi S, Roujol S, Akçakaya M, Kawaji K, Foppa M, Heydari B (2015). Accelerated cardiac MR stress perfusion with radial sampling after physical exercise with an MR-compatible supine bicycle ergometer. Magn Reson Med.

[22] Sharif B, Dharmakumar R, LaBounty T, Arsanjani R, Shufelt C, Thomson L (2014). Towards elimination of the dark-rim artifact in first-pass myocardial perfusion MRI: Removing Gibbs ringing effects using optimized radial imaging. Magn Reson Med.

[23] Chen L, Adluru G, Schabel MC, McGann CJ, DiBella EVR (2012). Myocardial perfusion MRI with an undersampled 3D stack-of-stars sequence. Med Phys.

[24] Fair MJ, Gatehouse P, DiBella EV, Chen L, Wage R, Firmin D (2016). An extended 3D whole-heart myocardial first-pass perfusion sequence: alternate-cycle views with isotropic and high-resolution imaging. J Cardiovasc Magn Reson.

[25] Fair MJ, Gatehouse PD, Chen L, Wage R, DiBella EVR, Firmin DN (2016). 3D first-pass myocardial perfusion stack-of-stars imaging using balanced steady state free precession. Proc Intl Soc Mag Reson Med.

[26] Mendes JK, Adluru G, Likhite D, Fair MJ, Gatehouse PD, Tian Y (2020). Quantitative 3D myocardial perfusion with an efficient arterial input function. Magn Reson Med.

[27] Shin T, Nayak KS, Santos JM, Nishimura DG, Hu BS, McConnell MV (2013). NThree-dimensional first-pass myocardial perfusion MRI using a stack-of-spirals acquisition. Magn Reson Med.

[28] Manka R, Jahnke C, Kozerke S, Vitanis V, Crelier G, Gebker R (2011). Dynamic 3-Dimensional Stress Cardiac Magnetic Resonance Perfusion ImagingDetection of Coronary Artery Disease and Volumetry of Myocardial Hypoenhancement Before and After Coronary Stenting. J Am Coll Cardiol.

[29] Manka R, Paetsch I, Kozerke S, Moccetti M, Hoffmann R, Schroeder J (2012). Whole-heart dynamic three-dimensional magnetic resonance perfusion imaging for the detection of coronary artery disease defined by fractional flow reserve: determination of volumetric myocardial ischaemic burden and coronary lesion location. Eur Heart J.

[30] Jogiya R, Morton G, Silva KD, Reyes E, Hachamovitch R, Kozerke S (2014). Ischemic Burden by Three-dimensional Myocardial Perfusion Cardiovascular Magnetic Resonance: Comparison with Myocardial Perfusion Scintigraphy. Circ Cardiovasc Imaging.

[31] Chung CS, Karamanoglu M, Kovács SJ (2004). Duration of diastole and its phases as a function of heart rate during supine bicycle exercise. Am J Physiol - Heart Circ Physiol.

[32] Fair MJC Development of whole-heart myocardial perfusion magnetic resonance imaging.

[33] Di Bella EVR, Parker DL, Sinusas AJ (2005). On the dark rim artifact in dynamic contrast-enhanced MRI myocardial perfusion studies. Magn Reson Med.

[34] Storey P, Chen Q, Li W, Edelman RR, Prasad PV (2002). Band artifacts due to bulk motion. Magn Reson Med.

[35] Fair M, Gatehouse P, Firmin D (2015). Through-plane dark-rim artefacts in 3D first-pass myocardial perfusion. J Cardiovasc Magn Reson.

[36] Fair MJ, Gatehouse PD, DiBella EVR, Firmin DN (2017). An analysis of radial sequence parameters in myocardial first-pass perfusion for optimised imaging. Proc Intl Soc Mag Reson Med.

